# VDAC Modulation of Cancer Metabolism: Advances and Therapeutic Challenges

**DOI:** 10.3389/fphys.2021.742839

**Published:** 2021-09-29

**Authors:** Kareem A. Heslop, Veronica Milesi, Eduardo N. Maldonado

**Affiliations:** ^1^Department of Drug Discovery and Biomedical Sciences, Medical University of South Carolina, Charleston, SC, United States; ^2^Facultad de Ciencias Exactas, Instituto de Estudios Inmunológicos y Fisiopatológicos (IIFP), UNLP, CONICET, CIC PBA, La Plata, Argentina; ^3^Hollings Cancer Center, Medical University of South Carolina, Charleston, SC, United States

**Keywords:** cancer, glycolysis, metabolic flexibility, metabolic reprogramming, metabolism, mitochondria, voltage dependent anion channels, Warburg

## Abstract

Most anionic metabolites including respiratory substrates, glycolytic adenosine triphosphate (ATP), and small cations that enter mitochondria, and mitochondrial ATP moving to the cytosol, cross the outer mitochondrial membrane (OMM) through voltage dependent anion channels (VDAC). The closed states of VDAC block the passage of anionic metabolites, and increase the flux of small cations, including calcium. Consequently, physiological or pharmacological regulation of VDAC opening, by conditioning the magnitude of both anion and cation fluxes, is a major contributor to mitochondrial metabolism. Tumor cells display a pro-proliferative Warburg phenotype characterized by enhanced aerobic glycolysis in the presence of partial suppression of mitochondrial metabolism. The heterogeneous and flexible metabolic traits of most human tumors render cells able to adapt to the constantly changing energetic and biosynthetic demands by switching between predominantly glycolytic or oxidative phenotypes. Here, we describe the biological consequences of changes in the conformational state of VDAC for cancer metabolism, the mechanisms by which VDAC-openers promote cancer cell death, and the advantages of VDAC opening as a valuable pharmacological target. Particular emphasis is given to the endogenous regulation of VDAC by free tubulin and the effects of VDAC-tubulin antagonists in cancer cells. Because of its function and location, VDAC operates as a switch to turn-off mitochondrial metabolism (closed state) and increase aerobic glycolysis (pro-Warburg), or to turn-on mitochondrial metabolism (open state) and decrease glycolysis (anti-Warburg). A better understanding of the role of VDAC regulation in tumor progression is relevant both for cancer biology and for developing novel cancer chemotherapies.

## Introduction

The relative contribution of aerobic glycolysis and oxidative phosphorylation (Oxphos) to overall ATP generation, determine cancer bioenergetics. Cancer metabolism, however, involves not only chemical reactions to cope with energy demands, but also those necessary to maintain anabolism and catabolism. The study of cancer metabolism preceded the discovery of oncogenes and tumor suppressors by approximately 50 years, becoming one of the oldest areas of research in cancer biology. A different metabolism in tumor cells, compared to non-proliferating cells, is regarded essential to develop and maintain malignant characteristics. A “reprogrammed metabolism,” considered by many a hallmark of cancer and observed quite generally across many types of tumor cells, is a major driver of tumor metabolism (Cerezo and Rocchi, 2020; Faubert et al., 2020). Metabolic reprogramming, induced by oncogenic mutations among other factors, refers to the enhancement or suppression of specific metabolic pathways in tumor cells that improve the cellular fitness required for rapid cell division. Despite its relevance for long-term cell survival, reprogramming is not sufficient to explain metabolic adaptations to fast changing demands. It is very likely that rapid metabolic changes be driven by fast acting mechanisms that modulate specific pathways or proteins not depending on reprogramming. In particular, regulation of the conductance of voltage dependent anion channels (VDAC), located in the outer mitochondrial membrane (OMM) will be described here as a mechanism potentially involved in fast metabolic responses.

Regardless of the relatively long history of research on cancer metabolism, the interest on the role of mitochondrial metabolism in tumors was limited until the end of the 20th century. A 2021 updated PubMed search using the words mitochondrial metabolism, or mitochondrial metabolism and cancer, showed only ~33 and 12% of the total publications during the period 1921–2000. The number of indexed publications for both topics exponentially increased in the last 2 decades. Even more strikingly, another search using the words VDAC and cancer, showed only ~3% of the total publications, reported from 1986 to 2000.

The relevance of mitochondria for cellular metabolism started to be unveiled in the early 20th century, when oxidations in the mitochondrial matrix and ATP synthesis, were identified as main functions of the organelle. However, a mechanistic explanation reconciling observation with a theory to explain the link between oxidation and ATP synthesis was lacking. The chemiosmotic hypothesis of Mitchell (1966) solved the problem by postulating a proton electrochemical gradient across the inner mitochondrial membrane (IMM) as the energy-rich intermediate of Oxphos (Mitchell and Moyle, 1967). Mitchell (1966) proposed that the flow of electrons through complexes of the electron transport chain (ETC) was coupled to the outward translocation of H^+^ across the IMM creating a proton motive force (Δp) used by the ATP synthase to synthesize ATP from adenosine diphosphate (ADP) and inorganic phosphate (Pi). The hypothesis of Mitchell (1966) was later confirmed by experimentation in mitochondria, chloroplasts, and bacteria.

A major trigger to research in cancer metabolism were the seminal findings made in the early 20th century by the German biochemist Otto Warburg, who showed in tumor slices and ascites cancer cells, that tumors produce more lactic acid than non-tumor cells even at physiological partial pressures of oxygen (Warburg et al., 1927; Warburg, 1930). This particular phenotype, called Warburg effect, is characterized by enhanced aerobic glycolysis. In tumor cells, relatively low cytosolic ATP/ADP ratios caused by a partial suppression of mitochondrial metabolism prevents glycolysis inhibition mediated by high ATP. Warburg (1956) even proposed that damaged mitochondria were the origin of cancer. According to his hypothesis, only those cells with irreversible but incomplete damage to respiration capable of increasing the conversion of glucose to lactic acid (fermentation) become cancerous. The provocative hypothesis of failing mitochondria as the origin of cancer was quickly challenged by Weinhouse (1956) and others, who demonstrated both high glycolysis and oxidative metabolism in cancer tissues. In fact, all tumors display a certain level of enhanced glycolysis that always coexists with functional mitochondria. Measurements of oxygen consumption, mitochondrial membrane potential (ΔΨ), 1,4-dihydronicotinamide adenine dinucleotide (NADH) production, and ATP generation, among other parameters, have confirmed that tumor mitochondria are metabolically active (Nakashima et al., 1984; Maldonado et al., 2010; Mathupala et al., 2010; Lim et al., 2011; Moreno-Sanchez et al., 2014; Singleterry et al., 2014). Variations in the relative contribution of mitochondrial ATP to cellular bioenergetics among different tumor types and even within the same tumor, suggest a dynamic regulation of oxidative metabolism.

In this review, we will describe the biological effects induced by changes in the conformational states of VDAC; how an increase of VDAC conductance reverses the Warburg phenotype and promotes cell death; and finally, the relevance of VDAC as a pharmacological target to develop novel cancer chemotherapies.

## VDAC Structure and Regulation of Conductance

The discovery of VDAC in mitochondrial extracts from *Paramecium tetraurelia*, followed by the identification in mammalian cells, opened a new avenue in the research and understanding of mitochondrial metabolism (Schein et al., 1976; Colombini, 1979). VDAC, comprising three isoforms, is a polypeptide of ~30kDa (VDAC 1 and 3: 280 amino acids; VDAC 2: 291 amino acids). In most mammalian cells, including cancer cells, VDAC1 and 2 are the most abundant isoforms, whereas VDAC3 is the least expressed, except for testis and spermatozoa (Sampson et al., 1997; De Pinto et al., 2010). VDAC β-barrels enclose an aqueous channel of ~3nm internal diameter in the open state, that allows the passage of molecules up to ~5kDa (Colombini, 1980, 2012; Mannella, 2021). The current consensus about VDAC structure shows differences with the originally proposed model by Colombini that was based on biochemical and functional data (Colombini, 2009, 2012). Structural studies using NMR and X-ray crystallography have shown VDAC1 and VDAC2 as a transmembrane β-barrel protein with 19 β-strands, mostly anti-parallel, except for strands 1 and 19. Both isoforms also have an N-terminal, α-helical region located within the pore (Ujwal et al., 2008; Hiller et al., 2010).

Voltage dependent anion channels inserted in non-polarized or weakly polarized membranes (close to 0mV), stays mostly in the high conductance open state. By contrast, positive or negative membrane potentials induce conformational changes to several lower conductance closed states (maximal at −45 or +45mV; Bowen et al., 1985; Colombini, 1989). Although, it is currently not possible to determine membrane potentials across the OMM in live cells, plausible theoretical approaches suggest the existence of polarization of the OMM in intact cells (Lemeshko, 2021). Moreover, the ~0.6 pH difference reported between the cytosol and the mitochondrial intermembrane space (IMS) (Porcelli et al., 2005), corresponds to a −15–20mV potential. Since this value of membrane potential falls in the range in which reconstituted VDAC display a mild decrease in conductance (Zizi et al., 1998), it is theoretically possible that voltage actually contributes to the regulation of VDAC opening.

Voltage dependent anion channels are selective for anionic metabolites and small cations, as showed initially by Colombini, and later confirmed by other groups (Choudhary et al., 2010; Villinger et al., 2014; Colombini, 2016). The open state of VDAC allows the flux of anions, including most respiratory substrates, ATP^4−^, ADP^3−^, HPO4^2−^, phosphocreatine^2−^, and AMP, among others. In the closed state, VDAC favors a non-selective flux of cations including Na^+^, K^+^, and Ca^2+^ (Tan and Colombini, 2007; Colombini, 2012; Sander et al., 2021). VDAC closure, induced by voltage, increases the flux of Ca^2+^ up to 10-fold (Tan and Colombini, 2007). Moreover, the magnitude of Ca^2+^ flux through VDAC is influenced by the type and amount of each isoform present (only VDAC1 and 2 seem involved in Ca^2+^ signaling); post-translational modifications (phosphorylation and monoubiquitinylation); and possibly interactions with partner proteins including Bcl-xL and translocator protein (TSPO), among others (Sander et al., 2021). However, a higher flux of calcium through VDAC in mitochondria of intact cells does not necessarily correlates with an increase in the Ca^2+^ content in the matrix. After entering the IMS, Ca^2+^ still needs to be transported through the IMM by the mitochondrial calcium uniporter holocomplex (Fan et al., 2020). Thus, mitochondrial uptake of Ca^2+^ is subjected to multiple levels of regulation both at the OMM and the IMM. It remains to be determined experimentally if VDAC-mediated increase in the flux of Ca^2+^, actually influences Ca^2+^ content in the mitochondrial matrix modifying mitochondrial metabolism.

Overall, every physiological or pharmacological regulation of VDAC to induce a change from the open state to the closed states, reduces or increases the flux of negatively charged metabolic substrates and cations, respectively. Although gating and selectivity for VDAC1 and VDAC2 are very similar in different cell types, the detailed molecular determinants of voltage gating are still incompletely understood. A structural model proposes that the N-terminus of VDAC1 lying inside the pore, parallel to the wall, moves to the lumen blocking the passage of metabolites (Shuvo et al., 2016). Regardless of the mechanism controlling gating, the flux of polar metabolites through VDAC is determined mostly by their charge and size (Colombini, 1980, 2004).

Even though VDAC was initially considered constitutively open, like an “all-time open gateway,” subsequent research both *in vitro* and in intact cells, showed regulation of VDAC conductance by several endogenous molecules. VDAC conductance has been shown to be modulated by α/β tubulin heterodimers (Rostovtseva et al., 2008; Timohhina et al., 2009; Maldonado et al., 2013); hexokinase (Pastorino et al., 2002; Al Jamal, 2005); bcl2 family members (Tsujimoto and Shimizu, 2000); glutamate (Gincel et al., 2001); and NADH (Zizi et al., 1994). It has also been demonstrated that post-translational modifications, mainly phosphorylation by protein kinases, GSK3β, PKA, and protein kinase C epsilon (PKCε), blocks or inhibits association of VDAC with other proteins, such as Bax and tBid, and regulates VDAC opening (Heiden et al., 2001; Baines et al., 2007; Das et al., 2008). Moreover, PKA-dependent VDAC phosphorylation and GSK3β-mediated VDAC2 phosphorylation increase VDAC conductance and also the sensitivity to tubulin inhibition (Bera et al., 1995; Das et al., 2008; Sheldon et al., 2011). VDAC opening is also modulated by protein-protein interactions with actin, p53, mitochondrial creatine kinase, and alpha-synuclein, among others (Rovini, 2019; Kanwar et al., 2020).

Overall, the movement of metabolites through VDAC dynamically depends on the concentration gradient of each permeant molecule reaching the OMM, the electric field, the number of functional VDAC channels, the selectivity-permeability to a particular metabolite, and the open probability of the channel.

## VDAC Opening, Mitochondrial Metabolism, and Warburg Effect

**Figure 1:**
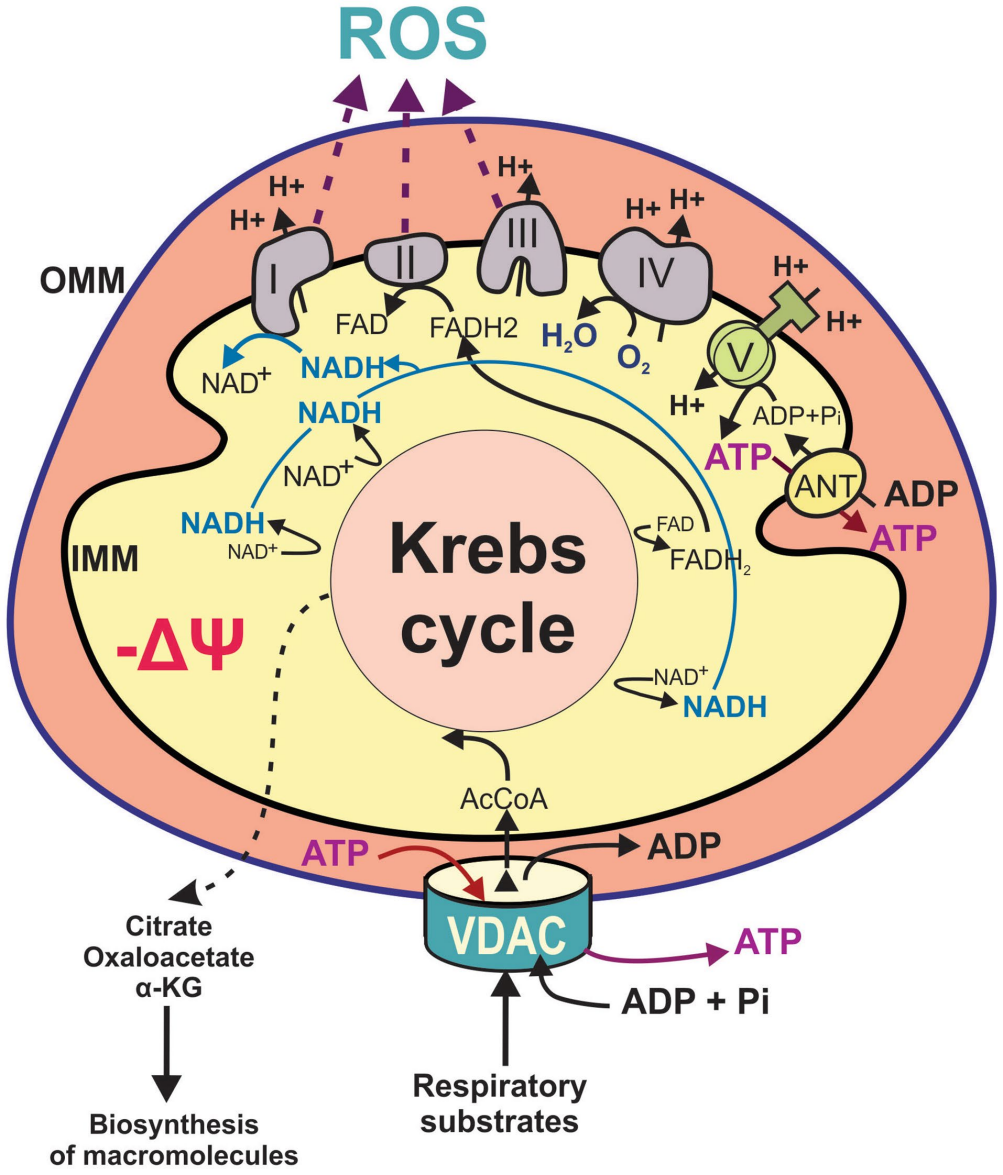
Main features of mitochondrial metabolism. Flux of metabolites including the respiratory substrates pyruvate, adenosine diphosphate (ADP), and inorganic phosphate (Pi) and certain amino acids, cross the outer mitochondrial membrane occurs through voltage dependent anion channels (VDAC). Catabolism of respiratory substrates in the Krebs cycle generates 1,4-dihydronicotinamide adenine dinucleotide (NADH) and dihydroflavine-adenine nucleotide (FADH_2_), which fuel the electron transport chain (ETC; complexes I–IV) to support oxidative phosphorylation. The Krebs cycle also produces metabolic intermediaries released to the cytosol for the biosynthesis of proteins and lipids. Proton pumping by the respiratory chain across inner mitochondrial membrane (IMM) generates mitochondrial ΔΨ. Protons moving back across MIM into the matrix drive ATP synthesis from ADP and Pi by the F_1_F_0_-ATP synthase (complex V). Mitochondrial ATP is exported from the matrix by the adenine nucleotide transporter (ANT) and released to the cytosol through VDAC. Flow of electrons through complexes I, II, and III also generates reactive oxygen species. AcCoA, acetyl CoA; α-KG, alpha ketoglutarate; IMM, inner mitochondrial membrane; OMM, outer mitochondrial membrane; and ROS, reactive oxygen species.

VDAC1, VDAC2, and VDAC3, at the interphase between mitochondria and cytosol, are strategically located to control the flux of metabolites and ATP entering or leaving mitochondria. At present, it is unclear if the flux of metabolites and nucleotides through VDAC is different among tumor cell types, and if it is isoform specific. To access the mitochondrial matrix, most anionic substrates that cross the OMM only through VDAC, are further transported through the IMM by several finely tuned specific carriers (Palmieri and Pierri, 2010). Pyruvate, fatty acids, and the amino acids glutamine (quantitatively the most important amino acid utilized by several tumors), glycine, serine, leucine, isoleucine, valine, and tryptophan, generate acetyl-coenzyme A (AcCoA) that fuels the Krebs (tricarboxylic acid) cycle. A cycle of oxidation generates NADH, and dihydroflavine-adenine nucleotide (FADH_2_), electron donors to the ETC. Electrons flowing through the ETC or respiratory chain, formed by complexes I-IV, reduce the final acceptor molecular O_2_ to H_2_O, while simultaneously generating single electron leaks from complexes I, II, and III to form the superoxide anion (O2^•-^), that is further converted into other reactive oxygen species (ROS) (Chance et al., 1979; Mailloux, 2020). The metabolic fitness of mitochondria in any cell type, also depends on the generation of ΔΨ formed when complexes I, III, and IV drive H^+^ translocation from the matrix to the IMS, where it generates a negative transmembrane ΔΨ and a ΔpH, both components of the proton motive force (Δp). Ultimately, Δp drives ATP synthesis from ADP and Pi by complex V (F_1_F_O_-ATP synthase): (Hoek et al., 1980; Nicholls and Ferguson, 2013). Newly synthesized ATP is transported to the cytosol through the adenine nucleotide translocator, located in the IMM, and exchanged for ADP in a 1:1 molar ratio to be finally released to the cytosol through VDAC (Klingenberg, 2008; Allouche et al., 2012). AMP, another adenine nucleotide, also cross the OMM through VDAC. Therefore, regulation of VDAC opening influences the ATP/AMP ratio, which in turn, modulates the activation of AMP-activated protein kinase (AMPK); (Colombini, 2016; Shevade et al., 2018). Thus, the magnitude of metabolite fluxes to support Oxphos and ATP synthesis, ultimately depends on VDAC conductance (Figure 1).

In general, approximately 95% of ATP in quiescent cells is produced by Oxphos, with the remaining 5% generated through the pay-off phase of glycolysis in the cytosol, and the succinyl-CoA ligase reaction of the Krebs cycle. Full mitochondrial oxidation of glucose generates ~32 moles of ATP, as estimated by different methods, compared to the 2 moles of ATP/mole of glucose during glycolysis (Brand, 2005). Although the total amount of mitochondrial ATP calculated considers the currently accepted proton stoichiometry for ATP synthesis, ATP/ADP-Pi exchange, respiration, and the malate/aspartate shuttle, the actual ATP yield could be less due to proton leak into the mitochondrial matrix (Brand, 2005; Rich and Marechal, 2010; Walker, 2013; Wikstrom et al., 2015). The relatively low ATP demand for cell division compared to the energy requirements for maintenance of cellular functions, mainly the activity of the Na^+^-K^+^ ATPase, suggests that ATP generation is not a limiting factor to sustain rapid cell proliferation (Kilburn et al., 1969; Schwenke et al., 1981; Veech et al., 2019; Seyfried et al., 2020). In non-proliferating cells, high VDAC conductance promotes an oxidative metabolism, generating cytosolic ATP/ADP ratios 50–100 times higher compared to mitochondria. High ATP-ADP in the cytosol inhibits phosphofructokinase-1 (PFK-1), a rate limiting step in the glycolytic pathway, among other possible mechanisms blocking glycolysis (Schwenke et al., 1981; Jenkins et al., 2011). The ATP-dependent inhibitory mechanisms, together with regulation of other pathways, may be key to explain the reciprocal dependence between mitochondrial metabolism and glycolysis in several tumor types. By contrast, a partial suppression of mitochondrial metabolism in cancer cells contributes to a low cytosolic ATP/ADP ratio; releasing the brake on glycolysis and favoring the Warburg phenotype. Compared to non-proliferating cells, cancer cells generate ~10–90% of total ATP by glycolysis (Nakashima et al., 1984; Griguer et al., 2005). Tumor cells also display “glucose avidity,” an increased uptake of glucose compared to non-proliferating cells. In clinical settings, the preferential incorporation of the radioactive glucose analog fluorodeoxyglucose by tumor cells is used in positron emission tomography (PET) to diagnose cancer (Zhu et al., 2011). A downside of PET scan that leads to false positives is the inability to distinguish between tumor cells and non-tumor cells with a high rate of glucose uptake, including tumor infiltrating lymphocytes.

The conformational open or closed state of VDAC, by regulating the flux of respiratory substrates, is a major determinant of cytosolic ATP/ADP ratios to favor or oppose the pro-proliferative Warburg phenotype. The predominance of open or closed conformational states of VDAC, not only determines the bioenergetics efficiency of mitochondria, but also the ability to contribute metabolic intermediaries to the synthesis of amino acids, fatty acids, nucleotides, cholesterol, glucose, and heme (Spinelli and Haigis, 2018). Rapidly dividing cells face a constant challenge to produce new macromolecules to approximately double the biomass before mitosis. In the Warburg metabolism, the major sources of carbon backbones are glucose, glutamine, and fatty acids. Similar to glycolytic intermediates, Krebs cycle intermediates are also used as precursors for biosynthesis of macromolecules. A continuous supply of precursors, sustained by anaplerotic pathways, “refills” the cycle with intermediates to replace those that have been already used for biosynthesis. Glutaminolysis, which produces α-ketoglutarate from glutamine, and pyruvate carboxylation, which produces oxaloacetate from glucose/pyruvate are major contributors to anaplerotic fluxes in cancer cells (Bott et al., 2019; Martins et al., 2020; Kiesel et al., 2021). Oxidation of the branched-chain amino acids (BCAAs) isoleucine and valine also provides an anaplerotic flux in some tumors. If glucose supply is sufficient for energy generation, glutamine-derived α-ketoglutarate and oxaloacetate are utilized for the synthesis of nonessential amino acids, whereas citrate exported to the cytosol, is converted into AcCoA and utilized for the synthesis of fatty acids, cholesterol, and amino acids. Glutamine is also a nitrogen donor in purine and pyrimidine synthesis, and a precursor for the synthesis of the antioxidant glutathione (Owen et al., 2002; Still and Yuneva, 2017; Li and Le, 2018; DeBerardinis and Chandel, 2020). Recently, one-carbon metabolism, a set of reactions that transfer one-carbon units (methyl groups) from serine and glycine, has been shown to be important for *de novo* synthesis of purines and thymidylate synthase in highly proliferative tumors (Meiser and Vazquez, 2016).

**Figure 2:**
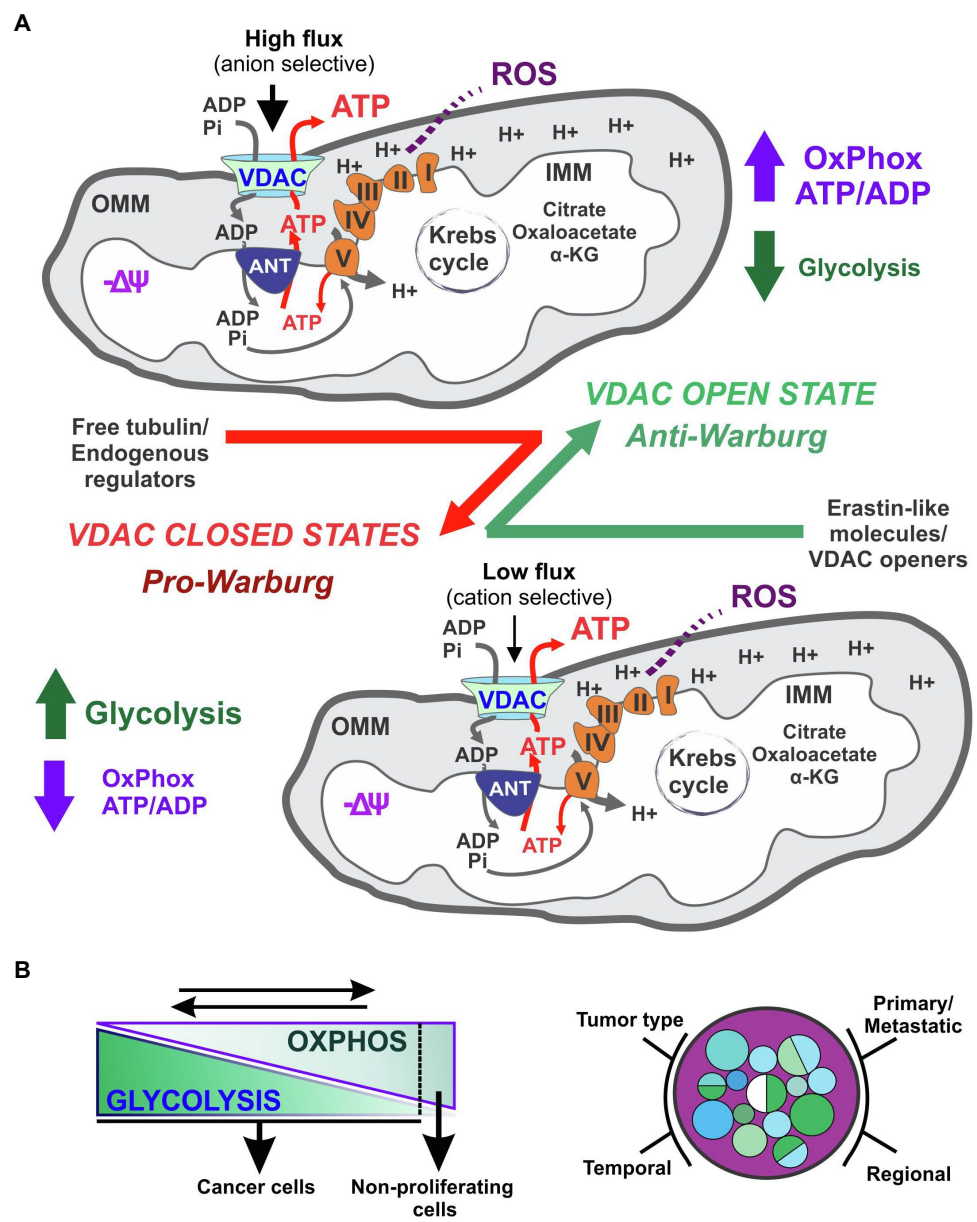
Voltage dependent anion channels regulation of cancer bioenergetics in metabolically flexible tumors. VDAC opening in cancer cells promotes oxidative metabolism and reverses the Warburg phenotype **(A)**. The contribution of glycolysis to cancer cell bioenergetics is influenced by the type of tumor, and regional differences, among other variables. A dynamic reversal of the Warburg phenotype influences cell proliferation **(B)**.

Overall, VDAC operates as a biological switch that, in the on-phase (open state), maximizes the flux of metabolites for optimal mitochondrial function, whereas during the off-phase (closed state), minimizes mitochondrial metabolism (Figure 2). Thus, regulation of only this channel has an amplifying effect on several intra and extra-mitochondrial pathways modulating cancer metabolism and bioenergetics. The dynamic changes in ATP and biosynthesis demands, ranging from seconds to minutes or even hours, imply the coexistence of both metabolic reprogramming and fast acting regulatory mechanisms. VDAC is, very likely, one of the rapidly adapting mechanisms that are responsive to interactions with other OMM and cytosolic proteins and soluble factors, along with transient or permanent posttranslational modifications. At present, it is unknown if metabolic reprogramming, that affects several mitochondria-related pathways, modulates VDAC conductance.

Beyond the role in mitochondrial metabolism, VDAC is also a prognostic biomarker for certain types of human cancer (Jozwiak et al., 2020; Wersall et al., 2021). High expression of VDAC1 has been associated with unfavorable outcomes in cancers from lung, head and neck, breast, and liver (Grills et al., 2011; Yang et al., 2019; Jozwiak et al., 2020). High transcript levels of VDAC2 in multiple tumors, including melanoma, epithelial thyroid tumors, and breast cancer have been reported in the cancer databases cBioportal,^1^ and the Human Protein Atlas.^2^ In head and neck cancer and in liver cancer, high expression of VDAC2 is also associated with a poor outcome. Interestingly, VDAC3 expression seems not to be of prognostic value for human cancer suggesting isoform specific effects on cell proliferation.

## Mitochondria, Tumor Metabolic Flexibility, and Tumor Heterogeneity

The term metabolic inflexibility, coined in the late 90’s, refers to inadequate responses of skeletal muscle to fuel changes in insulin-resistant obese patients (Kelley and Simoneau, 1994; Kelley et al., 1999). By contrast, metabolic flexibility alludes to the ability of muscle cells to switch from fatty acid to glucose oxidation. A “mitocentric” concept of nutrient metabolism describes the storage, utilization, and conversion of nutrients into other metabolites, as a critical process to monitor energy homeostasis (Trepanowski et al., 2011; Gambardella et al., 2020; Motori et al., 2020). Research to identify the molecular origins of metabolic flexibility has focused mostly on the interplay between glucose and fatty acids, and/or the aberrant production of the fatty acid precursor, malonyl-CoA. Mitochondria, as an integral metabolic hub, are major contributors to metabolic flexibility. Similar to muscle cells, most cancer cells are metabolically flexible. The different quantitative contributions of mitochondria to cellular bioenergetics, together with genomic instability and differences in the microenvironment, are important determinants of tumor heterogeneity (Kuipers et al., 2017; Barcena-Varela and Lujambio, 2021; Wei et al., 2021). Tumor metabolic heterogeneity is increasingly recognized as a factor causing failures in cancer treatment (Gentric et al., 2017; Kim and DeBerardinis, 2019). Whether a tumor displays a predominantly glycolytic or oxidative metabolism depends on gene expression as well as temporary and long-term epigenetic stimuli. In addition, the dynamic relative contribution of glycolysis and Oxphos is influenced by the type of primary or metastatic tumor, intra-tumor regional differences, temporal variations in the energetic demands and availability of glucose, fatty acids, ketone bodies, and certain amino acids (Jose et al., 2011; Alam et al., 2016; Duraj et al., 2021; Figure 2).

A reciprocal dependence of mitochondrial metabolism and enhanced glycolysis has been shown in several cancer cell types under different experimental conditions, including hypoxia and limitations in the availability of nutrients. The magnitude of Oxphos inhibition during hypoxia is influenced by the cell type and duration of the hypoxic exposure. Prolonged hypoxia increases glycolysis in MCF-7 cells but not in HeLa cells, although, Oxphos is the predominant source of ATP in both cell types (Rodriguez-Enriquez et al., 2010). Interestingly, in solid tumors, the respiratory chain is still fully functional at oxygen levels as low as 0.5%, indicating that cancer cells exposed to <2% oxygen in rapidly growing and heterogeneously perfused tumors still produce ATP by Oxphos (Vaupel et al., 2001; McKeown, 2014). In some human cell models, hypoxia induced the synthesis of a C-terminal truncated form of VDAC1, with similar channel activity and voltage dependency as the full-length channel. Truncated VDAC1 was linked to an upregulation of both Oxphos and glycolysis, as well as to resistance to apoptosis (Brahimi-Horn et al., 2012; Brahimi-Horn and Mazure, 2014; Mazure, 2016; Cunha-de Padua et al., 2020). By contrast, knockout of VDAC1 in mouse embryonic fibroblasts (MEF) expressing oncogenic RAS, favors tumor development in mice by promoting metabolic reprogramming (Brahimi-Horn et al., 2015). Beyond hypoxia, under conditions that decrease pyruvate oxidation in the Krebs cycle, mitochondria from tumor cells adapt to oxidize more glutamine as an energy source sustaining tumor growth both through aerobic glycolysis and Oxphos (Mullen et al., 2012). Nutrient availability not only influences tumor growth but also induces a switch from aerobic glycolysis to Oxphos in lymphoma and breast cancer cell lines cultured in glucose-free media (Smolkova et al., 2010; Robinson et al., 2012). If access to glucose and glutamine is limited, tumor cells adapt to utilize instead, lactate, methionine, arginine, cysteine, asparagine, leucine, acetate, and even lipids and proteins from the microenvironment to cope with the energy demands (Kreis et al., 1980; Clavell et al., 1986; Scott et al., 2000; Commisso et al., 2013; Kennedy et al., 2013; Comerford et al., 2014; Keenan and Chi, 2015). Overall, tumors display several metabolic alternatives to support continuous cell division regardless of unfavorable environmental conditions.

## VDAC as a Pharmacological Target

**Figure 3:**
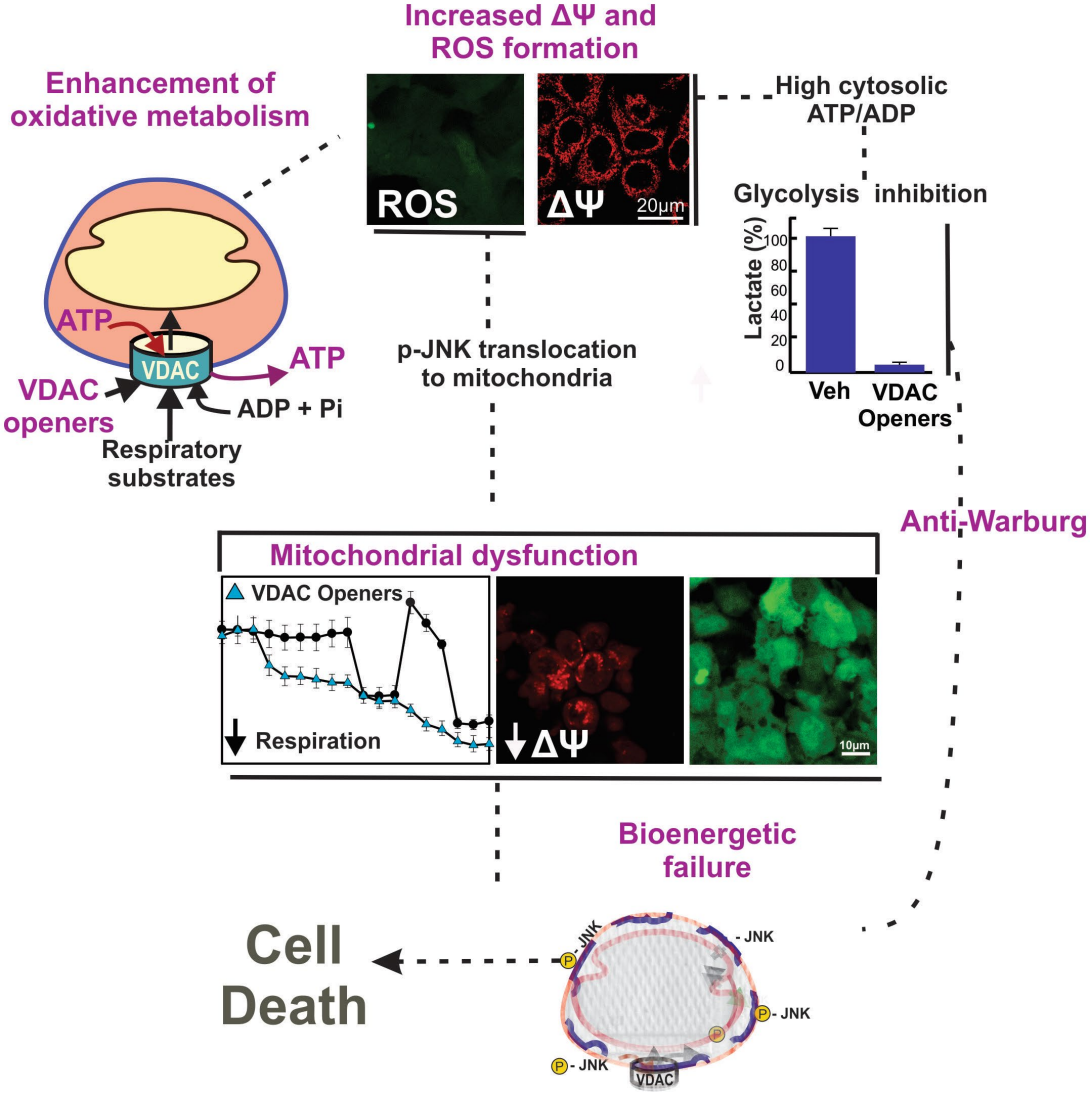
Working model of a two-hit mechanism of cell death induced by VDAC opening. VDAC-tubulin antagonists and potentially other VDAC openers increase the flux of metabolites into and out of mitochondria. VDAC opening increases mitochondrial metabolism, promotes high cytosolic ATP/ADP ratios, increases oxygen consumption, and decreases glycolysis (anti-Warburg). VDAC opening also increases mitochondrial ROS formation causing oxidative stress and mitochondrial dysfunction. Images are from confocal fluorescence microscopy of SNU-449 hepatocarcinoma cells loaded with chloromethyl-20,70–dichlorodihydrofluorescein diacetate (ROS), and tetramethyl rhodamine methyl ester (ΔΨ).

Conventional chemotherapeutic agents promote cell death or arrest cell proliferation by blocking DNA synthesis and replication, inhibiting specific enzymes or receptors, or by destabilizing or stabilizing microtubules. By contrast, “metabolic” approaches for cancer treatment have mostly focused on the inhibition of glycolysis. Only more recently, mitochondria and mitochondrial metabolism emerged as a source of targets to prevent or slow tumor progression (Adachi et al., 2004; Doherty and Cleveland, 2013; Bhat et al., 2015; Weinberg and Chandel, 2015; Fang and Maldonado, 2018; Curcio et al., 2021). In general, a restriction of mitochondrial oxidation of substrates decreases the amount of each intermediary released to the cytosol for the biosynthesis of macromolecules. By contrast, enhancement of mitochondrial metabolism leads to increased oxidation of substrates and ATP generation, increased ROS formation with subsequent oxidative stress, and reversal of the Warburg phenotype. These two ways of modulating mitochondrial metabolism decrease cell proliferation and promote cell death (Figure 3).

A remarkable and unique feature of VDAC is the ability to globally control mitochondrial metabolism by simply increasing or decreasing the conductance. In a broad sense, VDAC is a “first-step” in mitochondrial metabolism. Although, mitochondrial bioenergetics is regulated at multiple levels, VDAC opening in cancer cells leads to three major biological effects: maximization of full oxidation because of augmented entry of substrates into mitochondria; subsequent decrease in glycolysis due to a high cytosolic ATP/ADP ratio promoted by maximum generation of mitochondrial ATP; and increased formation of ROS following the enhanced activity of the *ETC*. Particularly relevant for cancer metabolism, VDAC has been shown to also serve as a docking site for a group of cytosolic proteins including hexokinase-II and the Bcl-2 family of proteins. While VDAC1 is considered a proapoptotic protein and VDAC2 seems to be anti-apoptotic, some controversy still remains about the role of VDAC for apoptotic cell death (Camara et al., 2017; Shoshan-Barmatz et al., 2017; Chin et al., 2018). Regardless, because of the obvious relevance of apoptosis for tumor growth, VDAC1 has emerged as a candidate to develop VDAC1-targeting molecules. Several peptides (mastoparan, mitoparan, and TEAM-VP); an oligonucleotide (G3139); molecules of unrelated structures (dicyclohexylcarbodiimide, estradiol, among several others); and more recently a miRNA (miR-7), have been reported to target VDAC and be pro or anti-apoptotic. These different molecules either regulate VDAC1 activity, expression, oligomerization, and interaction with HK-II or posttranslational modifications. An excellent review providing a brief description of compounds interacting mostly with VDAC1, have been published by De Pinto group (Magri et al., 2018).

Regardless of older and recent developments including a resolved NMR structure of VDAC1 with NADH bound (Bohm et al., 2020), and the identification of a cholesterol binding site (Budelier et al., 2017), drug discovery of VDAC modulators still faces major challenges. Despite the growing number of compounds that have an effect on VDAC, potential isoform specificities and identification of ligand binding sites for each of the reported molecules, are still unknown. An analysis of the structures of anti-cancer drugs acting on VDAC shows a lack of common structural motifs. In addition, a well-defined druggable binding site has not been yet identified. To add more complexity, and even when electrophysiology of VDAC inserted into lipid membranes is likely the best method currently available to study the effect of a molecule on VDAC conductance, the results may not be definitive. A different lipid composition of the artificial membranes compared to the OMM, lack of cytosolic soluble factors and protein interactions, limited knowledge about the effect of posttranslational modifications, potential artifacts introduced during the isolation and insertion of VDAC, as well as isoform specific responses, may lead to inconclusive results. Moreover, a compound of interest could eventually affect VDAC gating inserted into bilayers not only by interacting directly or indirectly with the channel, but also by altering the lipid bilayer surrounding the channel (Rostovtseva et al., 2020).

In the last 10 years, we showed that VDAC regulates mitochondrial metabolism in live cancer cells using a combination of knockdown strategies and confocal microscopy of ΔΨ and NADH, among other techniques. We also provided evidence that increased cytosolic free tubulin dynamically correlates with changes in ΔΨ, suggesting a direct effect of free tubulin on VDAC opening (Maldonado et al., 2013). Our initial findings about the effect of VDAC regulation by free tubulin on mitochondrial metabolism in intact cells were published shortly after dimeric α/β tubulin was shown to block VDAC conductance in lipid bilayers and in isolated mitochondria (Rostovtseva et al., 2008). We also showed that VDAC1 and 2 isolated from VDAC double-knockdown HepG2 cells in all combinations, inserted into lipid bilayers, were equally sensitive to tubulin inhibition. By contrast, VDAC3 was insensitive at tubulin concentrations even 5-fold higher than those used to inhibit VDAC1 and 2 (Maldonado et al., 2013). In parallel with those experiments, we showed that the blockage of the inhibitory effect of tubulin on VDAC by erastin, increased mitochondrial metabolism (Maldonado et al., 2013). Erastin is a VDAC 1 and 2-binding molecule identified in a synthetic lethal chemical screening shown to induce non-apoptotic cell death (Dolma et al., 2003). Interestingly, cell death induced by erastin was blocked by antioxidants but not prevented by pan-caspase inhibitors, suggesting that erastin-induced cell death was ROS-dependent (Yagoda et al., 2007). The effect of erastin on mitochondrial metabolism was independent of the inhibition of the cystine/glutamine antiporter system x_c_^−^, another known target of erastin (unpublished). More recently, using a cell-based high throughput screen, we identified a series of erastin-like compounds that enhance mitochondrial metabolism, promote oxidative stress leading to mitochondrial dysfunction, and decrease glycolysis as measured by lactate release (anti-Warburg; Wright et al., 2001; DeHart et al., 2018a,b). Lately, we showed that mitochondrial dysfunction promoted by erastin/erastin-like molecules was mediated by a ROS-dependent translocation of activated JNK to mitochondria (Heslop et al., 2019). In summary, the VDAC–tubulin interaction represents a new pharmacological target to turn a pro-proliferative phenotype into a cytotoxic, mitochondrial-dependent pro-oxidant metabolism. Based on our studies on VDAC regulation in cancer, we have proposed that VDAC opening is a pro-oxidant anti-Warburg switch that promote cancer cell death (Maldonado, 2017; Fang and Maldonado, 2018; Heslop et al., 2019).

Overall, pharmacologically-induced VDAC opening, as achieved by reversal of the inhibitory effect of tubulin on VDAC, triggers two distinct and nearly simultaneous effects: the increase of mitochondrial metabolism and Oxphos with subsequent decrease of glycolysis (anti-Warburg), and the increase in ROS formation causing oxidative stress (Figure 3). Because of the metabolic heterogeneity of tumors, it is possible that the adverse effects of ROS accumulation and glycolysis inhibition on cell survival and proliferation be different among cells. A VDAC-dependent increase in ROS production would be likely more detrimental for highly glycolytic cells, constitutively not exposed to high levels of mitochondrial ROS. Conversely, the reversal of the Warburg effect would damage more those highly glycolytic cells that survive oxidative stress and continue proliferating, or low glycolytic cells with a presumably constitutively higher basal level of ROS. The combination of reversal of Warburg metabolism and oxidative stress by erastin-like compounds caused cell death to human hepatocarcinoma cell lines in culture and slowed tumor growth in a xenograft model of Huh7 hepatocarcinoma cells (DeHart et al., 2018a,b).

An intriguing possibility is that VDAC be implicated also in controlling metabolic fluxes in cancer stem cells (CSC), also called stem-like cancer cells, tumor-initiating cells, or cancer-initiating cells. CSC comprising 1–2% of total cells in most types of cancer, have been found in breast, lung, colon, brain, head and neck, prostate and liver tumors, among others (Batlle and Clevers, 2017). Because of the capabilities for self-renewal, tumorigenesis, invasion, and migration, CSC are associated with high risk of metastasis and relapses after chemotherapy. CSC display either a more oxidative or a more glycolytic profile, are plastic, undergo phenotypic transitions, and depending on the tumor of origin, differentiate into non-stem tumor cells to sustain tumor growth (Fonseca et al., 2017; Gupta et al., 2019). An interesting study showed that the interaction between VDAC2 and a subunit of the phosphofructokinase 1 tetramer regulates glucose metabolism and modulates the phenotypic reprogramming of glioma stem cells (Zhou et al., 2018). This first work on the potential role of VDAC as a glycolytic regulator of the phenotype transition between CSC and non-stem cancer cells is opening new avenues to study VDAC as a potential therapeutic target. Although, a perspective about the role of mitochondria-tubulin interactions in the regulation of mitochondrial structure and function in CSCs has recently been reviewed, there is no actual evidence that the VDAC-tubulin interaction or VDAC opening modulates CSC plasticity and proliferation (Kim and Cheong, 2020).

## Concluding Remarks

Research on different aspects of cancer bioenergetics and cancer metabolism clearly showed that tumors are metabolically heterogeneous, and that many tumors, if not all, are metabolically flexible. Moreover, the pro-proliferative Warburg phenotype can be reversed by increasing oxidative metabolism. In that regard, VDAC opening is an attractive mechanism to target pharmacologically, because it serves the dual purpose of increasing mitochondrial metabolism and ROS formation, while at the same time decreasing glycolysis. The dual action of VDAC openers as anti-Warburg and promoters of oxidative stress, represent a two-hit mechanism to induce cell death. An advantage of VDAC as a target relies on the fact that regulation of only one protein has an amplifying effect, both on mitochondrial function and subsequently on biosynthetic reactions occurring in the cytosol, which depend on mitochondrial intermediaries. As it was developed here, the understanding of endogenous mechanisms regulating VDAC conformational states is relevant for cancer biology and for potential pharmacological applications. The search for drugs promoting VDAC opening directly, or indirectly by blocking endogenous regulators like is the case for VDAC-tubulin, open a new era in the development of novel metabolism-oriented cancer chemotherapy.

## Author Contributions

KH contributed with the search for updated literature, compilation of sources, writing and figures preparation. VM contributed with writing and preparation of Figure 2. EM organized the structure of the review, intervened in all the steps of the review preparation, contributed to the writing, and made final adjustments to the text and figures. All authors contributed to the article and approved the submitted version.

## Funding

This work was supported by NIH grant NCI R01CA184456 and SCTR Pilot Project UL1 TR001450-SCTR to EM; and the Abney Foundation Fellowship from MUSC Hollings Cancer Center to KH.

## Conflict of Interest

The authors declare that the research was conducted in the absence of any commercial or financial relationships that could be construed as a potential conflict of interest.

## Publisher’s Note

All claims expressed in this article are solely those of the authors and do not necessarily represent those of their affiliated organizations, or those of the publisher, the editors and the reviewers. Any product that may be evaluated in this article, or claim that may be made by its manufacturer, is not guaranteed or endorsed by the publisher.

## Footnotes

^1^ http://www.cbioportal.org

^2^ http://www.proteinatlas.org
